# Self-management of non-communicable diseases in low- and middle-income countries: A scoping review

**DOI:** 10.1371/journal.pone.0219141

**Published:** 2019-07-03

**Authors:** Jason Hearn, Isaac Ssinabulya, Jeremy I. Schwartz, Ann R. Akiteng, Heather J. Ross, Joseph A. Cafazzo

**Affiliations:** 1 Centre for Global eHealth Innovation, Techna Institute, University Health Network, Toronto, ON, Canada; 2 Institute of Biomaterials and Biomedical Engineering, University of Toronto, Toronto, ON, Canada; 3 Uganda Heart Institute, Mulago Hospital, Kampala, Uganda; 4 Uganda Initiative for Integrated Management of Non-Communicable Diseases, Kampala, Uganda; 5 Section of General Internal Medicine, Yale University School of Medicine, New Haven, CT, United States of America; 6 Ted Rogers Centre for Heart Research, Peter Munk Cardiac Centre, University Health Network, Toronto, ON, Canada; 7 Department of Medicine, University of Toronto, Toronto, ON, Canada; 8 Institute of Health Policy, Management and Evaluation, Dalla Lana School of Public Health, University of Toronto, Toronto, ON, Canada; The George Institute for Global Health, UNSW, AUSTRALIA

## Abstract

**Background:**

The prevalence of non-communicable diseases (NCDs) is rising in low- and middle-income countries (LMICs). Self-management, which enables patients to better manage their health, presents a potentially-scalable means of mitigating the growing burden of NCDs in LMICs. Though the effectiveness of self-management interventions in high-income countries is well-documented, the use of these strategies in LMICs has yet to be thoroughly summarized.

**Objective:**

The purpose of this scoping review is to summarize the nature and effectiveness of past interventions that have enabled the self-management of NCDs in LMICs.

**Methods:**

Using the scoping review methodology proposed by Arksey and O’Malley, PubMed was searched for relevant articles published between January 2007 and December 2018. The implemented search strategy comprised three major themes: self-management, NCDs and LMICs.

**Results:**

Thirty-six original research articles were selected for inclusion. The selected studies largely focused on the self-management of diabetes (N = 21), hypertension (N = 7) and heart failure (N = 5). Most interventions involved the use of short message service (SMS, N = 17) or phone calls (N = 12), while others incorporated educational sessions (N = 10) or the deployment of medical devices (N = 4). The interventions were generally effective and often led to improvements in physiologic indicators, patient self-care and/or patient quality of life. However, the studies emphasized results in small populations, with little indication of future scaling of the intervention. Furthermore, the results indicate a need for further research into the self-management of cardiovascular diseases, as well as for the co-management of diabetes and cardiovascular disease.

**Conclusions:**

Self-management appears to be an effective means of improving health outcomes in LMICs. Future strategies should include patients and clinicians in all stages of design and development, allowing for a focus on long-term sustainability, scalability and interoperability of the intervention in the target setting.

## Introduction

As a result of an ongoing epidemiological transition, low- and middle-incomes countries (LMICs) are currently experiencing a double burden of both infectious (i.e. communicable) and non-communicable diseases (NCDs) [1–3]. Though infectious illnesses have historically received the bulk of attention and resources in LMICs, nearly three quarters of global NCD deaths now occur in these countries [4]. This rise in NCD prevalence has led to various socioeconomic challenges in the affected LMICs, namely overloaded clinics, increased healthcare spending, and reduced economic productivity as a result of disability and death [5–7]. In considering these challenges, it is clear that there is a current need for creative interventions that will mitigate the growing global NCD burden and its associated challenges.

One form of intervention that has been of particular interest in recent years involves improving the *self-management* of individuals living with NCDs. Self-management encompasses any intervention that enables patients to better manage their health condition(s) on a daily basis. This includes technological interventions–such as smartphone applications [8], medical device deployment [9], and telemonitoring systems [10], as well as non-technological interventions–such as educational materials [11], in-person training sessions [12], and social support [13]. Though the prevalence and effectiveness of self-management interventions in high-income countries is well-documented [14–16], the degree to which these systems have been studied in LMICs remains poorly understood. Given the rising NCD prevalence, high clinical volume and healthcare resource constraints in LMICs, self-management presents an attractive means of mitigating the global NCD burden. By engaging patients directly, these interventions have the potential to improve NCD management in a scalable manner, thus reducing the burden of care currently incurred by overloaded clinics. To better understand how self-management can be harnessed to combat the global burden of NCDs moving forward, a thorough understanding of past interventions that have been deployed in LMICs is of great importance. Accordingly, a scoping review was performed to summarize the nature and effectiveness of NCD self-management interventions that have been deployed in LMICs.

## Methods

### Study design

Using the methodological framework proposed by Arksey and O’Malley [17], a scoping review was performed to assess the following research question: *What is known from the existing literature about the effectiveness of techniques that enable the self-management of NCDs in LMICs*? To ensure comprehensiveness and proper reporting, the scoping review was also guided by the checklist (see S1 Appendix) and flowsheet presented in the Preferred Reporting Items for Systematic reviews and Meta-Analyses extension for Scoping Reviews (PRISMA-ScR) [18].

### Search strategy

To identify relevant studies, a search strategy was devised to encapsulate the three aspects of the research question: self-management, NCDs and LMICs. The developed search strategy is summarized in Table 1. Using the established strategy, the investigators searched PubMed for articles that contained at least one search term from each column in Table 1, and that were published in a twelve-year period ranging from January 1, 2007 to December 1, 2018 (see exact search strategy used on PubMed in S2 Appendix). Through a preliminary analysis of the literature, it was concluded that the selected date range would capture the majority of solutions attempted since the epidemiologic shift from infectious diseases to NCDs in LMICs. A manual reference list review was also performed to extract additional citations that were missed in the initial search.

**Table 1:** Summary of search terms used for each dimension of the scoping review.

Self-management	NCDs	LMICs
self-management, remote management, SMS, short message service, text message, telemonitoring, telephone monitoring, phone-based, mobile technology, mobile health, mHealth, patient-centered	non-communicable disease, NCD, heart failure, diabetes, asthma, cardiovascular disease, stroke, heart attack, chronic respiratory, chronic pulmonary disease, chronic disease, treatment adherence	low-income, middle-income, LMIC, Africa, Asia, South America, poverty, developing country

### Inclusion and exclusion criteria

The inclusion criteria used in study selection were as follows: original research papers focused on the self-management of NCDs in LMICs, articles available in English, and articles that examined the direct effect of self-management on health outcomes. The authors excluded reviews, protocols and abstracts, as well as studies performed in HICs (as per the World Bank classification system). It should be noted that while review papers were excluded from the scoping review, they were used for reference list review to widen the reach of the search.

### Study selection

The authors first assessed titles and abstracts for relevance based on the established inclusion and exclusion criteria. Articles considered to be relevant based on the first pass of the PubMed database were exported into the Mendeley Desktop v1.19.2 (Elsevier, Amsterdam) reference management software. In the second phase, full-text manuscripts were used to further assess the relevance of the exported articles. Reference lists of the exported articles (including review papers) were also examined for relevant studies that were omitted in the initial search. New papers that emerged in this reference list review were added to the scoping review and included in the final synthesis if they met the inclusion criteria. Articles that met the inclusion criteria in the second phase were tagged for the subsequent data extraction phase, whereas those failing to meet the criteria were given a tag indicating the basis for their exclusion.

### Data extraction

A standardized table was then used to extract pertinent information from the articles tagged for inclusion. For each article, the table included the primary author, year of publication, targeted NCD(s), targeted country (or countries), study duration, sample size, description of the intervention and control groups, and key outcomes. Sample size was charted as the number of patients in each study group that were maintained for follow-up and thus, contributed to the health outcome measures. Given the scope of the study, key outcomes were considered to be any direct effects of the implemented intervention on a physiologic, quality of life or self-care metric.

## Results

### Overview of scoping review

**Fig 1:**
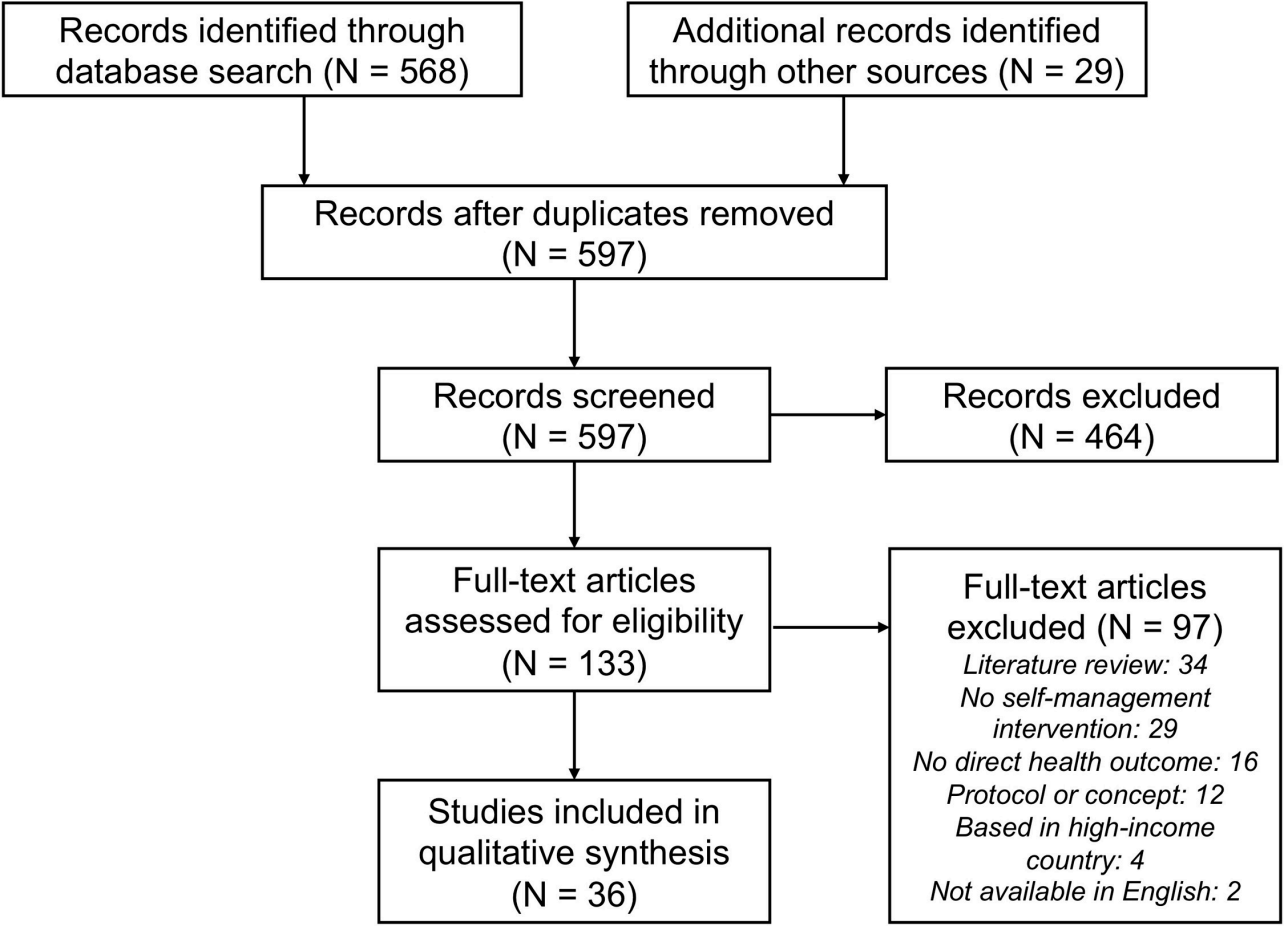
Summary of study selection process.

The initial search of the PubMed database returned 568 articles. Through analysis of these search results, including an additional 29 articles that emerged in the reference list review, 133 articles were deemed sufficiently relevant to warrant consideration of the full-text article. In reviewing the remaining articles, a total of 36 studies were selected for inclusion in the scoping review [19–54]. S3 Appendix presents a charted summary of the articles selected for inclusion. Studies not included in the data synthesis phase were generally excluded because they were literature reviews (which were kept for reference review purposes) (N = 34), they did not involve a self-management intervention (N = 29), they did not report a direct health outcome of an implemented self-management intervention (N = 16), or they were a protocol or concept for future work (N = 12). The study selection process is summarized in the PRISMA-ScR flowsheet [18] presented in Fig 1.

**Fig 2:**
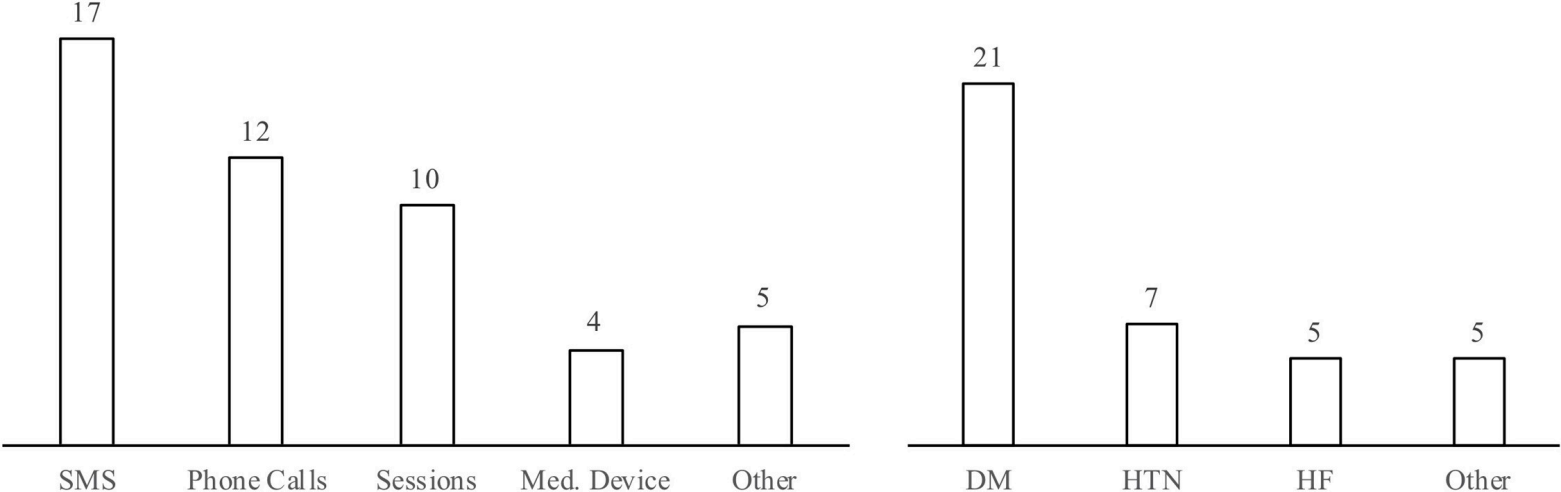
Modalities (left) and targeted conditions (right) of past interventions in LMICs.

In examining the results of the scoping review, it was determined that most of the identified interventions involved short message service (SMS, N = 17) or phone calls (N = 12), while a smaller proportion included educational sessions (N = 10) or the deployment of medical devices (N = 4). With regard to specific medical conditions, interventions were found to focus on diabetes mellitus (DM, N = 21), hypertension (HTN, N = 7) and heart failure (HF, N = 5) (Fig 2).

### Diabetes and prediabetes self-management interventions

In the area of DM self-management, several interventions have been piloted in LMICs. Past research studies have shown the potential for SMS interventions to improve various aspects of patient health. For example, SMS-based educational campaigns have been shown to improve haemoglobin A1c levels [29], fasting blood sugar levels and mean body mass index [39] in the Iranian DM population. Moreover, SMS systems targeted at reinforcing adherence behaviours have led to improvements in dietary habits amongst Filipino DM patients [50], as well as glycemic outcomes and treatment adherence amongst individuals with DM in India [48]. SMS interventions have also been shown to reduce incidence of type 2 DM amongst both Indian [43] and Chinese [52] men with impaired glucose tolerance. It should be noted that in the latter case amongst Chinese patients, the authors reported a significant reduction in incidence of type 2 DM at 12 months follow-up, but no significant difference after 24 months [52]. In a study that took place across three countries (Democratic Republic of Congo, Cambodia and Philippines), Van Olmen et al. reported no significant improvement in haemoglobin A1c levels when comparing an intervention group receiving educational SMS messages to a control group [51].

Phone calls have also been shown to be an effective means of improving self-management amongst DM patients in LMICs. In 2014, Piette et al. piloted an interactive voice response (IVR) system amongst DM and HTN patients living in Bolivia. The system would automatically call the patients weekly, ask them questions regarding their health status, symptoms and self-care behaviours, and subsequently provide tailored self-management education based on the provided responses. Though only half of the IVR calls were completed by the patients, the system led to improvements in perceived health status and medication adherence [41]. In a later study, the same research group tested the added benefit of a “CarePartner”–a family member or friend that would receive a summary of the feedback provided to the patient and suggestions for supporting the patient’s self-care. In the study, Piette et al. found that patients with a CarePartner were significantly more likely to report excellent health outcomes and less likely to report days in bed due to illness [40]. In Pakistan, Shahid et al. had success in calling DM patients twice per month to discuss self-care behaviours, reporting improvements in following a diet plan and in normalizing haemoglobin A1c levels [47].

Essien et al. encouraged Nigerian DM patients to attend 12 guideline-based teaching sessions over a six-month period, and saw a significant reduction in haemoglobin A1c levels when compared to a control group [27]. Debussche et al. implemented quarterly, peer-led educational sessions amongst DM patients in Mali, and found a significant improvement in haemoglobin A1c levels when comparing the intervention and control groups [24]. A research group in Guatemala had trained DM educators visit patients in their homes to deliver a culturally-appropriate, six-part curriculum. The single-group study saw a significant decrease in haemoglobin A1c levels from baseline to 12 months follow-up [28]. Two further research groups used frontline healthcare workers to deliver contextual self-management support to individuals with impaired glucose tolerance living in Philippines [34] and Brazil [25]. Both articles reported improvements in various aspects of self-management, as well as slight reductions in haemoglobin A1c levels, when comparing values at baseline and end-of-study follow-up.

The remaining DM interventions involved either medical device deployment or multimodal systems comprising several different methods of engagement. Namjoo Nasab et al. deployed a system wherein patients would attend four 90-minute educational sessions, and receive weekly phone calls from a trained nurse to discuss adherence to their self-care behaviours. At the end of the study period, the intervention group demonstrated significant improvements in fasting blood sugar and various DM self-management scores, when compared to a control group [37]. Investigators in Mexico established a system where patients received disease-specific information through a peer-led group educational session, as well as subsequent care management from a multidisciplinary healthcare team. A subset of the enrolled patients was also provided with a smart glucometer, test strips and a smartphone to assess the added benefit of device deployment. The investigators found that both the main group and the subgroup demonstrated significant reductions in haemoglobin A1c levels, but that the difference between the two intervention groups was insignificant [20]. Guo et al. deployed glucometers and test strips amongst Chinese DM patients, with a subgroup of patients having the stipulation that they could only keep the device if their haemoglobin A1c level decreased relative to their baseline value. Notably, the intervention group with the stipulation demonstrated a significant reduction in haemoglobin A1c levels when compared to both the control group and the intervention group without the stipulation [30]. Another DM-related study in India deployed a three-pronged remote management system that featured a counselling session, weekly educational SMS messages and follow-up calls from the investigator every three weeks. Patients in the intervention group reported significant reductions in average stress scores when compared to the control arm [38]. Lastly, a research group in Thailand deployed a system comprising email, SMS and a website to encourage self-care behaviours and goal setting, and reported significant improvements in haemoglobin A1c levels [53].

### Hypertension and prehypertension self-management interventions

In addition to the aforementioned interventions by Piette et al. that sought to improve both DM and HTN self-management in Bolivia [40,41], several systems have been piloted with the goal of improving HTN self-management, in particular. In 2012, Piette et al. deployed a HTN-specific intervention in Honduras and Mexico, where they provided patients with a home blood pressure monitor, as well as weekly automated calls pertaining to self-monitoring and behaviour changes. In the study, the intervention group demonstrated a decrease in mean systolic blood pressure when compared to the control group–a result that was further accentuated amongst a subset of patients with “high information needs” [42]. In South Africa, Bobrow et al. used SMS messages to motivate drug adherence amongst, as well as to provide HTN-specific education to, patients treated with high blood pressure. A subset of the intervention group was also given the ability to respond to the SMS messages with a “please call me” request. Both the main intervention group and the subgroup demonstrated reductions in systolic blood pressure when compared to the control group, however, the differences between the two intervention groups were insignificant [22]. In a second South African study using SMS messages to convey disease-specific knowledge and healthy lifestyle suggestions to HTN patients, the research team reported improvements in self-reported behaviour changes but no significant differences in overall health knowledge [31]. Lastly, Rubinstein et al. deployed a system comprising monthly motivational counselling calls and weekly SMS messages relating to diet and physical activity amongst a group of prehypertensive patients living in Guatemala and Peru. Though the intervention group failed to demonstrate significant improvements in blood pressure, those with access to the system did show a significant reduction in body weight and intake of foods high in fat and sugar [45].

### Heart failure self-management interventions

Self-management interventions for HF have been investigated in LMICs since as early as 2007, when Ramachandran et al. reported on a multi-pronged intervention that was piloted in India. In the study, patients in the intervention group received interactive sessions regarding HF and its management, as well as access to a “telephonic helpline” to reinforce the teachings and to modify medication dosages. Those using the system showed significant improvements in both quality of life and functional capacity when compared to a control group [44]. In 2009, a system was piloted in Brazil in which nurses would call HF patients twice per month to discuss recent complications, their cause, and recommendations on how they could have been managed (or even avoided) with drug treatment and/or lifestyle alterations. Despite the relatively small sample size (N = 40), there was a significant reduction in hospitalizations in the intervention group when compared to a control group [21]. Also in Brazil in 2011, Domingues et al. examined the efficacy of in-hospital educational sessions and post-discharge telemonitoring in improving self-care knowledge, but found that little additional benefit resulted from the telephone contact [26]. In 2013, a research group in Iran piloted a HF intervention comprising a one-hour nurse-led educational session, followed by bimonthly telephone calls from a nurse to reinforce teachings, improve coping abilities and enhance self-care behaviours. Though the intervention and control groups did not differ in self-care scores at baseline, the intervention group reported significantly-higher scores at one, two and three months post-discharge [54]. Most recently, Siddharthan et al. investigated the feasibility of using illustrated educational booklets, which described HF with simple text and accompanying pictograms, to improve self-management amongst Ugandan HF patients. The intervention led to improvements in patient activation measure scores across various areas (e.g. HF-specific knowledge, treatment options, and prevention of adverse outcomes), and was found to have a significantly larger effect amongst patients of low socioeconomic status [49].

## Discussion

The presented review highlights the general effectiveness of NCD self-management interventions that have been deployed in LMICs. Past interventions have shown self-management to be an effective means of improving physiologic indicators (e.g. haemoglobin A1c, blood pressure), self-care knowledge and quality of life amongst individuals living with chronic illness in LMICs. Moreover, the general success of these interventions does not appear to be restricted to specific geographic factors–such as language, culture or type of healthcare system, as the review includes studies performed in 21 countries across three continents. Both technological and non-technological interventions have demonstrated promise in LMICs, however, the emphasis appears to have been placed on mobile phone-based interventions. Given the recent and significant growth of mobile phone usage in LMICs [55], this focus on mobile health systems is both appropriate and expected.

Amongst the selected studies, the most commonly-implemented types of interventions were those that made use of SMS and phone calls. The popularity of these two modes of communication makes practical sense, as they are usable on any mobile device (smartphone or non-smartphone) with a network connection. Individuals of low socioeconomic status within LMICs are those that are most susceptible to developing NCDs; thus, the compatibility of these systems with low-cost devices is imperative for accessibility by the target population. Though smartphone prevalence continues to rise globally [56], it remains insufficient to make smartphone applications appropriate for wide-scale self-management interventions in most LMICs. The harnessing of mobile devices also provides greater potential for scalability than interventions involving educational sessions and medical devices, which are limited by human resource requirements and prohibitive device costs, respectively. Scalability considerations are of particular importance in self-management interventions, which require large-scale deployment if they are to create an effective and lasting impact.

With regards to the NCD(s) of interest in the selected studies, over half of the interventions focused on the self-management of DM patients, whereas a reduced number of studies targeted HTN and HF. The focus on DM is certainly warranted, as 80% of individuals with DM reside in LMICs, the majority of whom remain undiagnosed [57]. Moreover, the literature suggests that DM self-management is currently subpar in certain LMICs [58]. That being said, cardiovascular disease (CVD) presents an arguably-larger burden in LMICs, both at the national and individual level. More than 80% of all CVD-related deaths occur in LMICs [59], and the anticipated economic toll of CVDs in LMICs is expected to reach $7.1 trillion between 2011 and 2030 (compared to $800 billion dollars expected for DM during the same time period) [6]. Given the immense and growing burden of heart disease in LMICs, there appears to be a need for further research into the self-management of CVDs, including HTN and HF. Moreover, given the strong link between DM and CVD [60], the two conditions should not be treated in isolation. Future interventions should target the co-management of both DM and CVD, as proper management of both conditions is necessary for the avoidance of adverse patient outcomes [61].

The review also revealed a limited number of interventions that failed to achieve significant improvements in patient outcomes. In reviewing studies that reported no effect on the primary outcome, four studies cited issues in study design and execution, such as insufficient sample size [48], a follow-up period ill-suited to the target population [26], low fidelity [45], contamination between study groups, failure to consider alternative indicators [51], and patients lost to follow-up [31,51]. With regards to the deployment of SMS interventions, two studies reported technological issues such as delivery failures and the inability to tailor messages to the needs of specific patients [31,51]. One study cited materials that were unsuitable to the study population as a main reason for their lack of an effect [31]. These studies highlight the potential challenges that can arise in the development and assessment of self-management interventions in LMICs.

Despite the promising results of past NCD self-management initiatives, several shortcomings emerge in the presented works. First and foremost, most studies report findings from pilot-level implementation, with no clear vision for the future scaling and societal integration of the intervention. This focus on outcomes in small populations–and not on long-term interoperability and scalability–is a common phenomenon in global health research, so much so that it has been termed “pilotitis” by mobile health researchers in recent years [62]. In future projects, designers should strive for sustainability and compatibility with existing services by including local healthcare organizations and governmental agencies at each phase in the design process. From the listed interventions, it is also clear that there is a lack of investigation regarding mobile health interventions for patients living in sub-Saharan Africa–an area with ongoing increases in both NCD burden [63] and the prevalence of mobile devices [56]. The majority of the mobile health interventions that have been deployed in LMICs have also required staff engagement at each touchpoint between the patient and the system, reducing the potential scalability of the interventions. Researchers could consider reducing this requirement in mobile health interventions by using patient-submitted data to algorithmically triage patients in terms of risk, similar to the IVR system implemented by Piette et al. in Bolivia [41]. By triaging patients in this manner, those determined to be low risk could be simply managed through automated messages, while patients deemed to be high risk could be put in contact with clinic staff. Lastly, very few of the studies reported an involvement of local patients and/or clinicians in the design and development of their respective interventions. As this inclusion has been shown to be imperative for the design of successful mobile health systems in high-income countries [8], patients and clinicians should be engaged similarly when designing interventions for use in LMICs.

This study must be viewed in light of some limitations. Firstly, the selected studies were not evaluated in terms of their methodological quality. This limitation is one that is inherent to most scoping reviews, as the review aims to simply “map *rapidly* the key concepts underpinning a research area and the sources and types of evidence available” [64]. The review also stops short of determining which types of interventions are most likely to result in positive patient outcomes. This limitation again resulted from the mapping focus of the scoping review methodology, as well as the fact that a wide variety of different interventions were found to be effective in the review. As the search was limited to articles published in English, a second limitation is that the review could have neglected relevant studies available in other languages. Due to time and resource constraints, the review was also limited to articles indexed by PubMed–a database which primarily includes articles published in academic journals. As information pertaining to global health initiatives is often disseminated in news articles, blog posts and other informal sources, this final limitation may have omitted relevant self-management interventions.

## Conclusion

The burden of NCDs in LMICs is immense and growing. Self-management interventions, which enable patients living with NCDs to better manage their condition(s), demonstrate a potential means of mitigating the myriad of socioeconomic challenges currently faced by LMICs. Though self-management systems have been widely studied in high-income countries, the effectiveness of these interventions in LMICs remains poorly understood. This scoping review provides a thorough overview of NCD self-management interventions that have been deployed in LMICs. A total of 36 studies, taking place in 21 countries across three continents, were selected for inclusion in the review. The results of the selected studies were generally positive, with the interventions often leading to improvements in physiologic indicators, patient self-care and/or patient quality of life. The most commonly-studied NCD was DM, and the bulk of the interventions were either SMS- or phone call-based. Further research is indicated in LMICs for the self-management of CVD, as well as for the co-management of DM and CVD. Moreover, future interventions should focus on long-term sustainability in lieu of outcomes in small populations, as well as the inclusion of local patients and clinicians in all aspects of design and development.

## Supporting information

S1 Appendix PRISMA-ScR checklist. (PDF)

S2 Appendix Exact search strategy used for PubMed database query. (PDF)

S3 Appendix Study characteristics for NCD self-management interventions deployed in LMICs, where BMI = body mass index; BP = blood pressure; CG = control group; DM = diabetes mellitus; HbA1c = haemoglobin A1c; HF = heart failure; HTN = hypertension; IG = intervention group; SMS = short message service. (DOCX)
