# Supplementary material for: Self-management of non-communicable diseases in low- and middle-income countries: A scoping review
Source: PLoS One. 2019 Jul 3;14(7):e0219141. doi: 10.1371/journal.pone.0219141 (PMC6608949; doi:10.1371/journal.pone.0219141)
Supplement: S2 Appendix — (PDF) [file pone.0219141.s002.pdf]

S2 Appendix. Exact search strategy used for PubMed database query

("remote management" OR "self-management" OR "SMS" OR "short message service" OR "text message" OR "telemonitoring" OR "telephone monitoring" OR "phone-based" OR "mobile technology" OR "mobile health" OR "mHealth" OR "patient-centered") AND ("non-communicable disease" OR "noncommunicable disease" OR "NCD" OR "heart failure" OR "diabetes" OR "asthma" OR "cardiovascular disease" OR "stroke" OR "heart attack" OR "chronic respiratory" OR "chronic pulmonary disease" OR "chronic disease" OR "treatment adherence") AND ("low-income" OR "middle-income" OR "LMIC" OR "Africa" OR "Asia" OR "South America" OR "poverty" OR "developing country") AND (("2007/01/01"[PDat] : "2018/12/01"[PDat]))
