# Supplementary material for: Self-management of non-communicable diseases in low- and middle-income countries: A scoping review
Source: PLoS One. 2019 Jul 3;14(7):e0219141. doi: 10.1371/journal.pone.0219141 (PMC6608949; doi:10.1371/journal.pone.0219141)
Supplement: S3 Appendix — (DOCX) [file pone.0219141.s003.docx]

S3 Appendix. Study characteristics for NCD self-management interventions deployed in LMICs, where BMI = body mass index; BP = blood pressure; CG = control group; DM = diabetes mellitus; HbA1c = haemoglobin A1c; HF = heart failure; HTN = hypertension; IG = intervention group; SMS = short message service

| Author,  *Year* | NCD,  *Country* | Duration, *Group Size* | Study Groups | Key Outcomes |
| --- | --- | --- | --- | --- |
| Abaza et al., *2017* [19] | DM,  *Egypt* | 3 months,  *CG – 39;*  *IG – 34* | *CG* – received booklet containing general instructions for DM management.  *IG* – in addition to CG care, received educational SMS messages, as well as SMS reminders to take tests and to record readings. | Change in HbA1c levels did not differ significantly between the two groups at 3 months follow-up. However, 16 IG patients achieved the targeted 1% drop in HbA1c, compared to only 6 in the CG. IG also demonstrated larger improvements in mean blood glucose levels and average body weight when compared to CG. |
| Anzaldo-Campos et al., *2016* [20] | DM,  *Mexico* | 10 months,  *CG – 89;*  *IG-A – 92; IG-B – 83* | *CG* – received standard care.  *IG-A* – in addition to CG care, received a combination of care management by healthcare workers and a peer-led educational group.  *IG-B –* in addition to IG-A care, received a smart glucose meter, test strips and a 3G-enabled smartphone. | IG-A and IG-B demonstrated HbA1c reductions of 2.6% and 3.0%, respectively, compared to -1.3% for CG. IG-A and  IG-B showed improved DM knowledge compared to CG. No statistical difference was found between IG-A and IG-B. |
| Bento et al., *2009* [21] | HF,  *Brazil* | 6 months,  *CG – 20;*  *IG – 20* | *CG* – received conventional assistance and monthly phone calls of administrative nature (i.e. no educational element). Calls typically lasted 5 minutes, and focused on recent complications and their causes.  *IG* – received conventional assistance and educational phone calls every 15 days. Calls typically lasted 20 minutes, and focused on recent complications, their causes and recommendations regarding drug treatment and lifestyle alterations. | CG required 22 hospitalizations, compared to only 5 for IG. 50% of CG and 10% of IG were hospitalized during the follow-up period. These results were found to be statistically significant, even when controlling for the age of the group members. |

| Bobrow et al.,  *2016* [22] | HTN, *South Africa* | 12 months,  *CG – 396;*  *IG-A – 406;*  *IG-B – 394* | *CG* – received standard care.  *IG-A –* in addition to CG care, received SMS messages to motivate drug adherence and to provide education regarding HTN.  *IG-B* – in addition to IG-A care, could also respond with “please call me” requests at no additional cost. | IG-A and IG-B demonstrated mean adjusted change in systolic BP compared with CG of -2.2 and -1.6 mmHg, respectively. Odds ratio for the proportion of participants with a BP < 140/90 mmHg were 1.42 and 1.41 for IG-A and IG-B, respectively, when compared with CG. |
| --- | --- | --- | --- | --- |
| Boroumand et al.,  *2016* [23] | Coronary artery disease,  *Iran* | 3 months,  *CG – 32;*  *IG – 32* | *CG* – received 6 SMS messages per week with general health content.  *IG* – received 6 SMS messages per week regarding cardiac-specific self-management behaviours, such as physical activity, diet, medication and symptom management. | Despite having no difference between the mean scores for cardiac self-efficacy of IG and CG at the outset, IG showed significantly higher values at 3 and 4 months after the start of the intervention. |
| Debussche et al., *2018* [24] | DM,  *Mali* | 12 months,  *CG – 75;*  *IG – 76* | *CG* – received conventional care, including individual counselling sessions.  *IG* – in addition to CG care, received peer-led education sessions every 3 months, covering themes such as cardiovascular risk management, food intake, exercise, and blood glucose and insulin management. | IG demonstrated a 1.05% decrease in HbA1c levels, which was significantly larger than the CG value of 0.15%. IG also demonstrated a significantly larger decrease in mean BMI and waist circumference when compared to CG. |
| do Valle  Nascimento et al., *2017* [25] | Pre-DM,  *Brazil* | 6 months,  *IG – 52* | *IG –* received DM self-management support, including both motivational interviewing and behavioural action planning, from trained community health agents during their monthly home visits. | IG reported improvements in physical activity, consumption of vegetables, medication adherence, mean low-density lipoprotein levels, and triglyceride levels when comparing baseline and 6 months follow-up. Patients also reported slight improvements in HbA1c, but no decreases in consumption of high-fat foods or sweets. |
| Domingues et al.,  *2011* [26] | HF, *Brazil* | 3 months,  *CG – 60;*  *IG – 46* | *CG* – received educational nursing intervention during hospitalization.  *IG* – in addition to CG care, received telephone monitoring after discharge. | Scores for HF and self-care knowledge were similar at baseline. After 3 months, both groups showed significant improvements, however, no difference was noted between groups. |

| Essien et al., *2017* [27] | DM, *Nigeria* | 6 months,  *CG – 51;*  *IG – 53* | *CG –* patients were encouraged to attend the existing teaching sessions. These sessions lacked structured framework and no educational materials were provided.  *IG –* patients were encouraged to attend 12 comprehensive, guideline-based teaching sessions over a 6-month period. | IG demonstrated a mean 6-month HbA1c of 8.4%, compared to 10.2% in CG. This difference was found to be statistically significant and was robust to adjustment for covariates. |
| --- | --- | --- | --- | --- |
| Flood et al.,  *2017* [28] | DM,  *Guatemala* | 12 months, *IG – 90* | *IG* – received 6 home visits conducted by a DM educator and based on a culturally- and linguistically-appropriate curriculum that was designed specifically for the rural Mayan populations. | IG achieved a significant decrease in HbA1c levels from baseline to 12 months follow-up. IG also achieved a significant decrease in systolic BP; however, the change in diastolic BP was not significant. |
| Goodarzi et al.,  *2012* [29] | DM, *Iran* | 12 weeks,  *CG – 38;*  *IG – 43* | *CG* – did not receive any educational SMS messages during the intervention period.  *IG –* received 4 educational SMS messages per week regarding diet, exercise, medication and symptom management. | IG demonstrated significant improvements in HbA1c, low-density lipoproteins, cholesterol, blood urea nitrogen, micro-albumin, DM knowledge and self-care efficacy, when compared to CG. |
| Guo et al., *2014* [30] | DM, *China* | 6 months,  *CG – 41;*  *IG-A – 40;*  *IG-B – 37* | *CG* – were not provided with glucometer or strips.  *IG-A* – provided with glucometers and strips, however, could only keep the glucometers if their HbA1c levels declined compared to baseline.  *IG-B –* provided with glucometers and strips. | IG-A had significant declines in HbA1c and medical costs compared with the baseline, while IG-B and CG had a decrease in HbA1c only. BMI did not change significantly in any group. There was a significant difference in HbA1c of IG-A compared to IG-B and CG. |
| Hacking et al., *2016* [31] | HTN, *South Africa* | 17 weeks,  *CG – 70;*  *IG – 76* | *CG –* received standard care.  *IG* – in addition to CG care, received 90 SMS messages throughout the study covering knowledge of HTN and healthy lifestyle suggestions. | No significant differences in overall health knowledge between IG and CG. IG showed increase in self-reported behavior changes, as well as a strong preference for the SMS messages. |
| Kamal et al., *2015* [32] | Stroke, *Pakistan* | 2 months,  *CG – 83;*  *IG – 79* | *CG –* received standard care.  *IG –* in addition to CG care, received 2 SMS messages per week containing personally-tailored medication reminders. | After 2 months, IG and CG had mean medication scores of 7.4 and 6.7, respectively, with an adjusted mean difference of 0.54. Mean diastolic BP in IG was also 2.6 mmHg lower than CG. |

| Khonsari et al.,  *2015* [33] | Acute coronary syndrome, *Malaysia* | 2 months,  *CG – 31;*  *IG – 31* | *CG –* received standard care.  *IG* – in addition to CG care, received automated SMS reminders before every intake of cardiac medication. | | IG showed higher medication adherence, while the risk of being low adherent among the CG was 4.09 higher than IG. IG also showed a higher heart function status compared to CG. | |
| --- | --- | --- | --- | --- | --- | --- |
| Ku et al., *2014* [34] | DM,  *Philippines* | 12 months,  *IG – 164* | *IG –* received contextual DM self-management education and support from trained local governmental personnel. The support system was adapted for the target population based on the local chronic care models, as well as the knowledge, attitudes and practices of local DM patients. | | IG reported significant improvements in DM knowledge, positive attitude, perceived self-management ability and medication adherence, when comparing baseline and 12 months follow-up. Reductions in HbA1c were also demonstrated in 60.4% of IG patients. | |
| Liu et al., *2008* [35] | Chronic obstructive pulmonary disease,  *China* | 3 months,  *CG – 24;*  *IG – 24* | *CG* – asked to take daily walking exercise.  *IG –* asked to walk at a speed controlled by the tempo of music coming from an application on their phone. The tempo was adjusted monthly based on test results. The duration of walking and symptom scores were recorded daily and sent to a website for storage and monitoring. | | IG showed significant improvements in incremental shuttle walk test distance, duration of endurance, inspiratory capacity and quality of life, while the changes in the CG values were either negative or statistically insignificant. | |
| Lv et al., *2012* [36] | Asthma, *China* | 12 weeks,  *CG – 14;*  *IG-A – 30;*  *IG-B – 27* | *CG –* received verbal asthma education from outpatient physician, including asthma facts, verbal and graphic interpretation of lung function results, medication actions and inhaler technique.  *IG-A –* in addition to CG care, received SMS messages about how to manage asthma at 10:00am and 8:00pm every day during the study. The content included classes of medication, proper device use, strategies to avoid triggers and how to handle attacks.  *IG-B –* in addition to CG care, received a free peak expiratory flow meter and were trained on proper use, what to do if reading fell in danger zones, how to keep an asthma diary, and how to adjust plan based on diary entries. | | IG-A and IG-B showed significant increases in the six-item perceived control of asthma score, with IG-A showing a greater change than IG-B. IG-A patients also had the highest Standard Asthma-Specific Quality of Life score and follow-up rate, although the change in perceived control of asthma was associated with the change in asthma-specific quality of life. All groups improved in forced expired volume in one second when compared to baseline. No differences were noted between the three groups in forced expired volume in one second or eosinophil/neutrophil counts in blood and sputum. | |
| Namjoo Nasab et al., *2017* [37] | DM,  *Iran* | 3 months,  *CG – 30;*  *IG – 30* | *CG* – received standard care.  *IG* – in addition to CG care, attended four 90-minute educational session, covering DM-specific topics such as risk factors, diet, physical activity and self-management behaviours. Patients also received weekly phone calls from a trained DM nurse, allowing for discussion about self-care behaviours. | IG demonstrated significant improvements in fasting blood sugar and various DM self-management scores, when compared to CG. Specifically, IG demonstrated significant improvements over CG in terms of glucose management, dietary control and physical activity scores. | |  |
| Patnaik et al., *2015* [38] | DM, *India* | 3 months,  *CG – 21;*  *IG – 34* | *CG* – received printed educational materials.  *IG* – received counseling with intense lifestyle education using both printed materials and computers. Patients were then called by the investigator every 3 weeks, and SMS messages were sent every week containing educational information. | At baseline, average stress scores in IG and CG were similar at 18.9. After 3 months, the IG mean score had dropped to 17.1, while the CG value increased to 20.7. A higher proportion of stress reduction was also noted in IG. | |  |
| Peimani et al.,  *2016* [39] | DM, *Iran* | 12 weeks,  *CG – 50;*  *IG-A – 50;*  *IG-B – 50* | *CG* – did not receive SMS messages.  *IG-A* – received tailored SMS messages, where 75% of the messages were based on the top two barriers to adherence that the participant had reported in the initial assessment.  *IG-B* – received non-tailored SMS messages, where random messages were sent regarding all potential barriers to adherence. | HbA1c level did not significantly change in any of the groups. A significant decline in fasting blood sugar and mean BMI was observed in IG-A and IG-B. These two groups also showed a significant increase in the Self-Care Inventory score and decreases in mean scores for the DM Self-Efficacy Scale and Diabetes Self-Care Barriers Assessment Scale for Older Adults. CG showed the opposite trend in each of the three self-care measures. | |  |
| Piette et al., *2016* [40] | DM; HTN,  *Bolivia* | 4 months,  *CG – 27;*  *IG – 45* | *CG* – received weekly interactive voice response calls, in which the system would ask questions regarding self-management and would subsequently provide tailored feedback based on the patient’s responses.  *IG* – in addition to CG care, patient provided a family member or friend (called CarePartner) that would receive a summary of the feedback provided to the patient and suggestions for supporting the patient’s self-care. | When compared to patients in CG, IG patients were significantly more likely to report excellent health outcomes and less likely to report days in bed due to illness. Call completion rates were higher for patients in the IG group. Amongst the subgroup of indigenous and low-literacy patients, those in IG were three times more likely than CG to complete calls with the interactive voice response system. | |  |
| Piette et al., *2014* [41] | DM; HTN,  *Bolivia* | 12 weeks;  *IG – 165* | *IG* – received weekly interactive voice response calls at times the patient indicated as convenient. The system used a tree-structured algorithm to assess patients’ self-management behaviors, perceived health status and symptoms. Based on this assessment, the system would then provide the patient with tailored self-management education and, if needed, would advise them to contact their doctor. | Likelihood that patients would report excellent, very good or good health increased during participation and was associated with improved medication adherence. Patient’s completed 51% of interactive voice response calls attempted in the twelve-week period. | |  |
| Piette et al., *2012* [42] | HTN, *Honduras; Mexico* | 6 weeks,  *CG – 92;*  *IG – 89* | *CG* – received BP results at baseline, HTN information, and usual healthcare.  *IG* – in addition to CG care, received a home BP monitor, as well as weekly automated monitoring and behavior change telephone calls. | IG showed a decrease in mean systolic BP 4.2 mmHg greater than CG. In the subgroup of IG with “high information needs”, mean systolic BP dropped by 8 mmHg. IG also reported fewer depressive symptoms, fewer medication problems, better general health and greater satisfaction with care, when compared to CG. | |  |
| Ramachandran et al., *2013* [43] | Pre-DM, *India* | 2 years,  *CG – 266;*  *IG – 271* | *CG* – received standard lifestyle modification advice at baseline.  *IG* – in addition to CG care, received SMS messages at frequent intervals regarding healthy lifestyle, the benefits of physical activity and diet and strategies to remain motivated in healthy habits. | Cumulative incidence of type 2 DM was lower in IG than in CG, with prevalences of 18% and 27%, respectively. No significant changes were found in BMI, waist circumference, BP or cholesterol. Adherence to diet was higher in IG, while adherence to physical activity did not differ between the two groups. | |  |
| Ramachandran et al., *2007* [44] | HF, *India* | 6 months,  *CG – 25;*  *IG – 25* | *CG* – received traditional care in HF clinic.  *IG* – in addition to CG care, received interactive sessions regarding the disease, drugs and self-management of fluid intake and diuretic dose, as well as a telephonic helpline to reinforce the information and to modify drug dosages. | IG showed a significant improvement in quality of life when compared to CG. IG also demonstrated improvement in the functional capacity measured by the six-minute walk test, while the CG regressed. There was no significant change in the number of hospital visits. | |  |

| Rubinstein et al.,  *2016* [45] | Pre-HTN, *Guatemala; Peru* | 12 months,  *CG – 287;*  *IG – 266* | *CG* – received standard care.  *IG* – in addition to CG care, received monthly motivational counselling calls and weekly personalised SMS messages about diet quality and physical activity. | IG showed no significant change in BP when compared to CG. IG did show a significant reduction in body weight and intake of high-fat/high-sugar food when compared to CG. IG also showed increase in the intake of fruits and vegetables. |
| --- | --- | --- | --- | --- |
| Sarfo et al.,  *2018* [46] | Stroke,  *Ghana* | 3 months,  *IG – 20* | *IG* – received a smartphone application (and a smartphone, if necessary) that delivers an individualized, goal-oriented exercise program. The 5-days-per-week program was monitored and progressively graduated by a remote therapist. Patients could also record videos of their daily exercises, which would be reviewed by the therapist and discussed in a weekly conference call. | IG demonstrated significant improvement in the Stroke Levity Scale score at both  1 and 3 months follow-up. IG also demonstrated notable improvements in the Montreal Cognitive Assessment, specifically at 3 months follow-up. |
| Shahid et al.,  *2015* [47] | DM, *Pakistan* | 4 months,  *CG – 220;*  *IG – 220* | *CG –* physically examined initially and after 4 months in the clinic*.*  *IG –* in addition to CG care, received calls on their mobile phones every 15 days regarding their self-monitoring of glucose, medication intake, physical activity and eating habits. | IG showed improvement in following diet plan, while CG had an insignificant increase. IG also demonstrated significant positive association with normalization of HbA1c levels, even when controlled for various covariates. |
| Shetty et al.,  *2011* [48] | DM, *India* | 12 months,  *CG – 66;*  *IG – 78* | *CG –* received standard care, with clinic reviews every 3 months.  *IG –* in addition to CG care, received SMS messages every 3 days as a reminder to strictly follow the regimen of dietary modification, physical activity and drug schedules. | The difference between HbA1c levels in IG and CG was insignificant, however, a significantly higher percentage of patients in IG showed better glycaemic outcomes. Adherence to medical nutrition therapy was improved in IG. |
| Siddharthan et al., *2016* [49] | HF, *Uganda* | 3 months,  *IG-A – 49;*  *IG-B – 44* | *IG-A –* based at a private clinic, received a patient-centered education tool that facilitates communication by describing diseases in a short, illustrated booklet with simple text and pictograms. The booklet was incorporated in outpatient visits, where it was distributed in a short appointment with a healthcare worker.  *IG-B –* received same care as IG-A, but were instead based at a public clinic. | Both groups showed improved patient activation measure scores regarding disease-specific knowledge, treatment options and prevention of exacerbations. The effect of the intervention was significantly larger among poorer patients. Patients noted that the materials were simple, that they had improved their knowledge of disease and increased communication with physicians. |
| Tamban et al.,  *2013* [50] | DM, *Philippines* | 6 months,  *CG – 36;*  *IG – 46* | *CG –* during regular visit to endocrinologist, received 15-minute educational session about diet and exercise, and were encouraged to follow up monthly.  *IG –* in addition to CG care, received 3 SMS messages per week regarding facts about proper diet or exercise, as well as a reminder to adhere to diet and exercise given by the educator. | Significant improvements were observed in mean number of meals per day, mean number of minutes per exercise and mean HbA1c, when comparing IG and CG. |
| Van Olmen et al., *2017* [51] | DM,  *Democratic Republic of Congo;*  *Cambodia;*  *Philippines* | 24 months,  *CG – 380;*  *IG - 401* | *CG* – received standard care, in addition to a mobile phone at the time of inclusion in the study.  *IG* – in addition to CG care, received SMS messages 2-to-6 times per week (depending on the study location), containing DM-specific information pertaining to the disease process, diet, physical activity, medications, blood glucose monitoring, and the identification of complications. | Proportion of participants with controlled HbA1c levels was 2.8% larger in IG, when compared to CG; however, this result was not statistically significant. The odds ratio of having controlled DM in IG was 1.1, after adjusting for baseline HbA1c level, sex, insulin treatment and participation in standard care. |
| Wong et al.,  *2013* [52] | Pre-DM, *China* | 24 months,  *CG – 29;*  *IG – 41* | *CG –* received standard care.  *IG –* in addition to CG care, received SMS messages regarding DM, lifestyle modification, social norms of how others appreciate lifestyle modification, and self-efficacy enhancing statements on how to control behavior. | Fewer patients developed DM in IG at 12 months when compared to CG. Relative risk of DM was 0.35 at 12 months and 0.62 at 24 months. When comparing IG and CG, a significant odds ratio of 0.04 was shown at 12 months and an insignificant ratio of 0.34 was shown at 24 months. |
| Wongrochananan et al., *2015* [53] | DM, *Thailand* | 3 months,  *CG – 30;*  *IG – 55* | *CG –* received weekly information about self-management of DM through email only.  *IG –* received an interactive multi-modality technology intervention, consisting of email, SMS and a website to provide four main functions: self-regulation, self-monitoring/assessment, social support, and reminders. Patients were encouraged to set goals for self-management by logging on to the website. | A significant difference in HbA1c level was demonstrated between IG and CG. Almost all physiological and biochemical parameters decreased in both groups, except for cholesterol and BMI in IG and HbA1c in CG, where increases were found. However, only the increase in HbA1c in CG was found to be significant. |
| Zamanzadeh et al., *2013* [54] | HF, *Iran* | 3 months,  *CG – 40;*  *IG – 38* | *CG –* received standard care.  *IG –* in addition to CG care, received a two-part intervention comprising a) a one-hour nurse-led HF education session personalized to the participant’s level of education and prior knowledge, and b) a post-discharge telephone follow-up to reiterate and review the information covered in the session, to improve the patient’s ability to cope, and to enhance self-care behaviors. | The two groups did not differ in self-care scores at baseline, however the scores were significantly higher in IG after 1, 2 and 3 months. IG demonstrated significant differences in self-care behaviors over the 3 months. |
